# Health outcomes, education, healthcare delivery and quality – 3057. Randomized, double blind comparative study to assess safety, efficacy with mometasone & formoterol versus fluticasone & formoterol dry powder inhaler (DPI) in the treatment of mild to moderate persistent asthma

**DOI:** 10.1186/1939-4551-6-S1-P225

**Published:** 2013-04-23

**Authors:** Sathish Chandra

**Affiliations:** 1 Department Of Community Medicine, Bangalore, India

## Background

Asthma is a problem worldwide, with an estimated 300 million affected individuals. Mometasone being newer drug has low systemic bioavailability, high glucocorticoid receptor affinity and modifies inflammatory mediators involved in the pathogenesis of asthma. Mometasone significantly improves PFT and symptom control in patients with asthma when used in combination with Formoterol. There are very few studies conducted to assess and compare the safety and efficacy of Mometasone group (Mometasone & Formoterol) versus Fluticasone group (Fluticasone & Formoterol). Hence the present study was undertaken to assess the safety and efficacy of Mometasone group versus Fluticasone group using DPI in patients with mild to moderate persistent asthma and also its effect on symptom control and frequency of rescue medication use.

## Methods

The present study was conducted in PMU, Bangalore during March 2011-2012. 60 patients were recruited in each arm based on inclusion and exclusion criteria. PFT was done pre and post bronchodilator with Salbutamol nebulization with Spirometry. Study medications were randomized, double blinded and were given for 12weeks & comparison was done to know safety, efficacy and frequency of rescue medication use before and after treatment in asthmatics. Statistical test -descriptive statistics, repeated ANOVA, Z- test, t- test.

## Results

Out of 60 patients in Fluticasone group, 11 developed adverse reactions- 4 developed recurrent URTI whereas in Mometasone group- 7 developed adverse reactions- 2 each developed recurrent URTI & hoarseness of voice. There was an overall improvement in lung function test (FVC, FEV1, FEV1/FVC, FEF25-75, PEF) between Mometasone and a Fluticasone group which was statistically significant when compared within the group (*P*=0.001), but was not significant when compared between the groups suggesting both are equally efficacious. There was a significant reduction in symptoms before and after treatment within the group, but the reduction was not statistically significant when compared between the groups suggesting both are equally efficacious. There was a significant reduction in dosage of rescue medication used from baseline to the end of 12weeks in Mometasone group compared to Fluticasone group (t value= 6.96; *P*= 0.001).

## Conclusions

Both Mometasone +Formoterol and Fluticasone+ Formoterol combinations were safe and equally efficacious in treating asthmatics.

